# Targeting Triple-Negative Breast Cancer with Momordicine-I for Therapeutic Gain in Preclinical Models

**DOI:** 10.3390/cancers17142342

**Published:** 2025-07-15

**Authors:** Kousik Kesh, Ellen T. Tran, Ruchi A. Patel, Cynthia X. Ma, Ratna B. Ray

**Affiliations:** 1 Department of Pathology, Saint Louis University, St. Louis, MO 63104, USAellen.tran.1@health.slu.edu (E.T.T.);; 2 Department of Medicine, Medical Oncology, Washington University in St. Louis, St. Louis, MO 63110, USA; cynthiaxma@wustl.edu; 3 The Alvin J. Siteman Cancer Center, Washington University School of Medicine, St. Louis, MO 63110, USA

**Keywords:** triple-negative breast cancer, momordicine-I, therapy, signaling pathway, macrophage polarization

## Abstract

***Background****:* TNBC patients respond poorly to chemotherapy, leading to high mortality rates and a worsening prognosis. Here, we investigated the effect of M-I on TNBC tumor growth suppression and its potential mechanisms. ***Methods****:* Signaling pathways were analyzed to study the effect of M-I on TNBC cells (human MDA-MB-231 and mouse 4T1). We used orthotopic mouse models to examine the anti-tumor efficacy of M-I. Tumor volume and the status of tumor-associated macrophages (TAMs) were assessed by qRT-PCR or FACS analysis. ***Results****:* We found a significant dose- and time-dependent inhibition of TNBC cell proliferation following treatment with M-I. Cell cycle analysis revealed a shortened S phase in M-I-treated cells and downregulation of AURKA, PLK1, CDC25c, CDK1, and cyclinB1. Furthermore, M-I treatment reduced the expression of pSTAT3, cyclinD1, and c-Myc in TNBC cells. To evaluate the anti-tumor efficacy of M-I, we employed orthotopic TNBC mouse models and observed a significant reduction in tumor growth without measurable toxicity. Next, we analyzed RNA from control and M-I-treated tumors to further assess the status of TAMs and observed a significant decrease in M2-like macrophages in the M-I-treated group. Immortalized bone marrow-derived mouse macrophages (iMacs) exposed to conditioned media (CM) of TNBC cells with or without M-I treatment indicated that the M-I treated CM of TNBC cells significantly reduce the M2phenotype in iMacs. Mechanistically, we found that M-I specifically targets the IL-4/MAPK signaling axis to reduce immunosuppressive M2 macrophage polarization. ***Conclusions****:* Our study reveals a novel mechanism by which M-I inhibits TNBC cell proliferation by regulating intracellular signaling and altering TAMs in the tumor microenvironment and highlights its potential as a promising candidate for TNBC therapy.

## Simple Summary

Triple-negative breast cancer (TNBC) is an aggressive subtype of breast cancer with limited treatment options and poor prognosis owing to the lack of targetable receptors. We recently identified momordicine-I (M-I), a bioactive metabolite in bitter melon, which displays anti-tumor activity in head and neck cancer with no measurable toxic effect. However, the anti-cancer efficacy of M-I in TNBC remains unknown. This study aimed to examine whether momordicine-I exhibits an anti-cancer effect on TNBC and to elucidate the underlying mechanisms. We found that M-I suppresses TNBC cell proliferation and significantly reduces tumor progression in TNBC mouse models. M-I suppressed TNBC cell-mediated immunosuppressive M2 macrophage polarization. To the best of our knowledge, this is the first report showing the anti-cancer efficacy of M-I in TNBC preclinical models and the potential underlying mechanism for tumor regression.

## 1. Introduction

Triple-negative breast cancer (TNBC) is one of the most aggressive and clinically challenging breast cancer subtypes and accounts for approximately 10–20% of all breast cancer cases. TNBC lacks estrogen receptor (ER), progesterone receptor (PR), and human epidermal growth factor receptor 2 (HER2) expression. It is disproportionately associated with younger patients, higher metastatic potential, and significantly poorer outcomes than hormone receptor-positive breast cancers [1,2]. The lack of ER, PR, and HER2 expression renders standard targeted therapies ineffective, leaving chemotherapy as the primary systemic treatment modality. Unfortunately, the effectiveness of chemotherapy is limited, frequently resulting in tumor recurrence and metastatic disease progression [3].

Alternative treatments using herbal agents against various cancers have become a growing field of research and have gained significant attention, owing to their exceptional therapeutic effects in cancer [4]. Phytochemicals and their derivatives from plant or microbial sources are promising alternative therapeutic options that have shown favorable results in preclinical studies [5,6,7,8]. We and others have shown potential anti-cancer effects of bitter melon (*Momordica charantia*) extract and its active components in preclinical models [5,9,10,11]. Bitter melon contains diverse secondary metabolite classes, including cucurbitane-type triterpenes; phenolic acids; flavonoids; essential oils; sterols; saponin; and primary metabolites, including fatty acids, amino acids, lectins, and some proteins [9,10,11]. Among these, momordicine-I (M-I), a bioactive cucurbitane-type triterpenoid isolated from *Momordica charantia*, has shown notable anti-cancer effects in head and neck cancer models through mechanisms involving the inhibition of several key signaling pathways and induction of cell death. M-I was found to influence the reprogramming immune environment in a head and neck cancer mouse model [12]. However, the anti-cancer effect of M-I in TNBC, particularly its impact on the tumor microenvironment (TME), has not yet been evaluated.

The TME, particularly the presence and phenotype of tumor-associated macrophages (TAMs), has gained attention as a critical contributor to tumor progression, metastasis, and therapeutic resistance. TAMs are highly plastic immune cells that can adopt various phenotypes depending on the stimuli within their microenvironment. Among these phenotypes, the M2 macrophage subtype is particularly implicated in tumor progression due to its pro-tumoral functions, including immunosuppression, promotion of angiogenesis, tissue remodeling, and metastasis facilitation [13]. Increased infiltration of M2-polarized macrophages has been correlated with worse prognosis and reduced survival rates in patients with TNBC, emphasizing the importance of targeting these cells as part of a therapeutic strategy [14].

Here, we demonstrate that M-I treatment suppresses TNBC cell proliferation and reduces tumor volume in TNBC mouse models. We found that conditioned media from TNBC cells enhances macrophages to the M2 phenotype, an effect that is abrogated when TNBC cells are treated with M-I. Our findings suggest that M-I treatment not only reduces tumor growth in preclinical models but could possibly reprogram TAMs, implicating its potential as a therapeutic candidate for TNBC.

## 2. Materials and Methods

### 2.1. Cell Culture and Momordicine-I (M-I) Treatment

TNBC human cell line MDA-MB-231 (RRID:CVCL_0062) was cultured in Dulbecco’s Modified Eagle’s Medium (DMEM) supplemented with 10% fetal bovine serum (FBS) and 1% penicillin–streptomycin at 37 °C in a humidified atmosphere with 5% CO_2_. The mouse TNBC 4T1 (RRID:CVCL_0125) cell line and immortalized bone marrow-derived macrophages (iMacs) were cultured in RPMI 1640 medium supplemented with 10% FBS. Momordicine-I (>98% purity) was purchased from Chemfaces (Cat. No.: CFN92076; Wuhan, Wuhan, China) and dissolved in DMSO, and cells were treated at the indicated concentrations as described previously [12].

### 2.2. Cell Viability Assay

TNBC human and mouse cells were seeded in a 96-well plate (5000 cells/well) and allowed to adhere for 24 h. Cells were treated with M-I at increasing concentrations (0–20 μM) for different time points. Cell viability assays were performed using a WST-8-based cell cytotoxicity assay according to the manufacturer’s protocol (Dojindo, Rockville, MD, USA) and expressed after normalization to untreated cells.

### 2.3. RNA Isolation and Quantitative Real-Time PCR

Total RNA was isolated using TRIzol reagent, and cDNA was synthesized using a random hexamer and Superscript III reverse transcriptase (Thermo Fisher Scientific, Waltham, MA, USA). Real-time quantitative PCR was performed using specific primers (Table 1) with a SYBR green-based detection system (Thermo Fisher Scientific). 18s rRNA was used as an endogenous control. Relative gene expression was measured using the 2^−ΔΔCT^ formula (ΔΔCT = ΔCT of the sample − ΔCT of the untreated control). Triplicate samples were used.

### 2.4. Western Blot Analysis

Cell lysates were prepared using 2XSDS sample buffer, and Western blot analysis was performed using specific antibodies to Cyclin D1 (1:500, Santa Cruz Biotechnology, Dallas, TX, USA, Cat# sc-20044, RRID:AB_627346), c-Myc (1:1000, Cell Signaling Technology, Danvers, MA, USA, CST-18583, RRID:AB_2895543), pSTAT3 (1:1000, Cell Signaling Technology, CST-9145 (Tyr705) (D3A7) RRID:AB_2491009), STAT3 (1:1000, Cell Signaling Technology, CST-30835 (D1B2), RRID:AB_2798995), pERK 1/2 (1:1000, Cell Signaling Technology, CST-4370, RRID:AB_2315112), and ERK 1/2 (1:1000, Cell Signaling Technology, CST-4696S). The HRP-conjugated anti-mouse or anti-rabbit secondary antibodies (1:2000) were obtained from Santa Cruz Biotechnology (RRID:SCR_008987). The membrane was reprobed with actin-HRP antibody (1:2500, SC-517582, RRID:AB_2833259) or tubulin-HRP antibody (1:1000, SC-23948, RRID:AB_628410) to compare protein loading in each lane.

### 2.5. Cell Cycle Analysis

MDA-MB-231 cells were cultured in three 60 mm plates in DMEM supplemented with 10% FBS. The cells were treated with the control (vehicle) or M-I. After 18 h of treatment, the cells were harvested by trypsinization, followed by fixation in 70% (*v*/*v*) ethanol at 4 °C overnight. The fixed cells were washed twice and resuspended in PBS containing 50 µg/mL propidium iodide (PI) and 0.1 mg/mL RNaseA. After incubation for 4 h at 4 °C in the dark, the cells were subjected to FACS analysis using a FACScan flow cytometer (BD Biosciences, San Jose, CA, USA). Data was analyzed using ModFit LT V4.1.7(Win) software (BD Biosciences, San Jose, CA, USA).

### 2.6. Animal Model

Female athymic nude (RRID:IMSR_ENV:HSD-069) or Balb/c mice (7 and 8 weeks of age) were purchased from Inotiv, Inc., West Lafayette, IN, USA. MDA-MB-231 cells (1 × 10^6^) or 4T1 cells (1.5 × 10^5^) containing 40% Matrigel were injected into the left mammary fat pads of nude or BALB/c mice. Once the tumors were palpable, mice were randomized into two groups (*n* = 5/group). Mice were treated daily with vehicle (control group) or M-I (30 mg/kg/day) (dissolved in 5% DMSO/95% of a 30% *w*:*v* Captisol solution—experimental group) by intraperitoneal (i.p.) injection. Body weight was measured once a week, tumor volume was measured using a slide caliper, and tumor volume was calculated using the following formula: 1/2 *L* × *W*^2^. At the end of the experiment, the mice were euthanized using a CO_2_ chamber (as approved in the protocol), and the tumors were collected for FACS analysis or snap-frozen in liquid nitrogen for further analysis.

### 2.7. Flow Cytometry for Tumor-Infiltrated Immune Cells

Mouse tumors were placed in RPMI before processing. Tumors were minced into small pieces, digested with collagenase IV at 37 °C for an hour in the presence of DNase 1, and passed through a 40 μm nylon filter for single-cell populations. For flow cytometry, the cells were stained with Live/Dead Aqua, CD45-Brilliant Violet 650™, F4/80-APC/Cyanine7, and CD206-PE. Flow cytometric analysis was performed using a BD LSRFortessa flow cytometer (BD Biosciences, San Jose, CA, USA) at the Saint Louis University Flow Cytometry Core Facility. The data were analyzed using FlowJo v10.10.0 software (Tree Star Inc., Ashland, OR, USA, RRID:SCR_008520). Antibodies used for immune cell analysis were all from BioLegend, San Diego, CA, USA: CD45 (BioLegend 1:400, RRID:AB_2564383), F4/80 (BioLegend 1:200, RRID:AB_893477), and CD206 (BioLegend 1:200, RRID:AB_10896421).

### 2.8. Statistical Analysis

Data are presented as mean ± standard deviation (SD). Statistical analyses were performed using Microsoft Excel (RRID:SCR_017294) or GraphPad Prism version 10.0 (RRID:SCR_002798). Differences between two groups were analyzed using Student’s *t* test. A *p*-value < 0.05 was considered statistically significant. The experiments were repeated at least in triplicate, and representative data are shown.

## 3. Results

### 3.1. M-I Treatment Reduces Proliferation of Triple-Negative Breast Cancer Cells In Vitro

**Figure 1:**
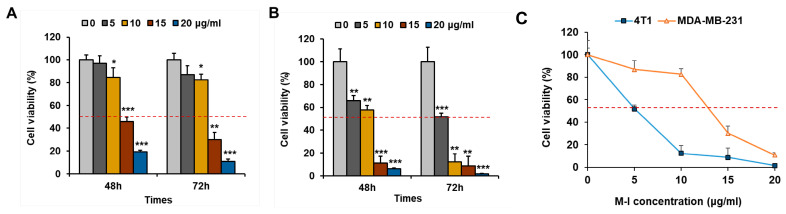
M-I treatment inhibits TNBC cell proliferation. TNBC human MDA-MB-231 (**A**) and mouse 4T1 (**B**) cells were treated with M-I at indicated concentrations and time points. Cell viability was measured using a Cell Counting Kit-8. Data represent mean ± SD (*n* = 3, * *p* < 0.05, ** *p* < 0.01, *** *p* < 0.001). (**C**) The IC50 doses of 4T1 and MDA-MB-231 treated with M-I for 72 h were 5 μg/mL or 10 μg/mL, respectively.

To investigate the anti-proliferative effects of M-I on TNBC cells, cell proliferation assays were performed. TNBC human MDA-MB-231 or mouse 4T1 cells were treated with M-I at different concentrations and time points. As shown in Figure 1A,B, M-I treatment significantly decreased the cell viability in a dose- and time-dependent manner. Treatment of 4T1 or MDA-MB-231 cells with M-I showed IC50 values of 5 μg/mL and 10 μg/mL, respectively (Figure 1C).

### 3.2. M-I Decreases Breast Cancer Growth Through Regulation of Cell Cycle Genes

**Figure 2:**
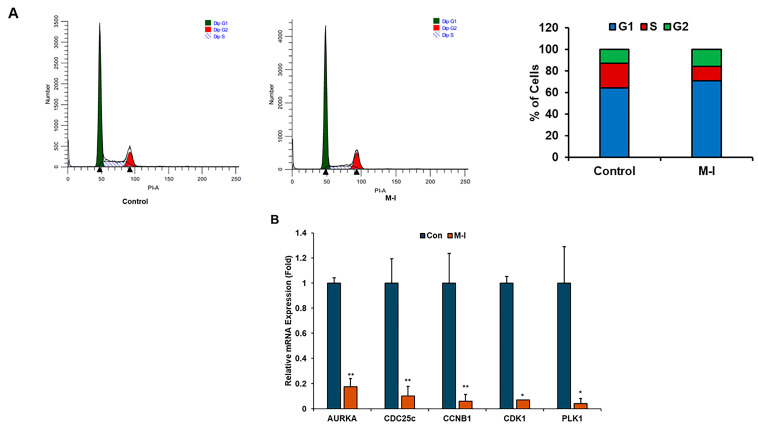
M-I impairs the cell cycle in TNBC cells. (**A**) Control or M-I-treated MDA-MB-231 cells were harvested, fixed, and stained with propidium iodide. DNA content was analyzed by flow cytometry. Results represent the cell population in the G1, S, and G2 phases of the cell cycle. The right panel shows the percentage of the cell population in different phases of the cell cycle. (**B**) Relative mRNA expression of AURKA, CDC25c, CCNB1, CDK1, and PLK1 in M-I-treated MDA-MB-231 cells was analyzed by qRT-PCR as compared to vehicle-treated cells. 18S rRNA was used as an internal control. Data represents SD (* *p* < 0.05, ** *p* < 0.01). Experiments were repeated three times with technical duplicates.

**Figure 3:**
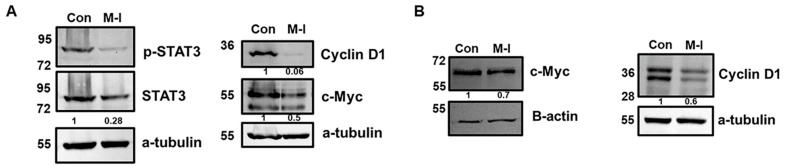
M-I reduces cell cycle regulatory proteins in TNBC cells. (**A**) Western blot analysis was performed on cell lysates from MDA-MB-231 cells treated with vehicle (Con) or M-I to examine the expression of key proteins involved in proliferation pathways. Representative Western blots showing reductions in pSTAT3, Cyclin D1, and c-Myc expression. (**B**) Representative Western blots showing reductions in Cyclin D1 and c-Myc expression in 4T1 cells treated with vehicle or M-I. An antibody against a-tubulin or beta-actin was used as an internal control for protein comparison. The values represent the indicated protein quantitation of Western blot band intensities normalized to the loading control using ImageJ software (version 1.54g), presented control as 1 (arbitrarily). The uncropped blots are shown in Supplementary Materials.

To investigate the mechanism of M-I-induced inhibition of TNBC cell proliferation, we performed flow cytometric analysis of DNA content. For this analysis, MDA-MB-231 cells were treated with vehicle or 15 μM of M-I for 18 h and stained with PI. Flow cytometric analysis revealed a shortened S phase in the cell cycle (Figure 2A). We observed that the number of actively dividing cells, represented by the S phase, was significantly declining in the M-I-treated group. The percentage of total cells in the S phase for the control was 23% and 14% in M-I treated cells (Figure 2A, bar graph). We next analyzed other molecules involved in the cell cycle. We also found that AURKA, PLK1, CDC25c, CDK1, and CCNB1 to be significantly downregulated (Figure 2B). Subsequently, we observed a significant downregulation of STAT3 phosphorylation, cyclin D1, and c-Myc expression in M-I-treated MDA-MB-231 and 4T1 cells (Figure 3A,B). Together, our results indicate that M-I suppressed TNBC cell proliferation mediated by regulation of cell cycle genes.

### 3.3. M-I Treatment Suppresses Tumor Progression in Orthotropic TNBC Mouse Models

**Figure 4:**
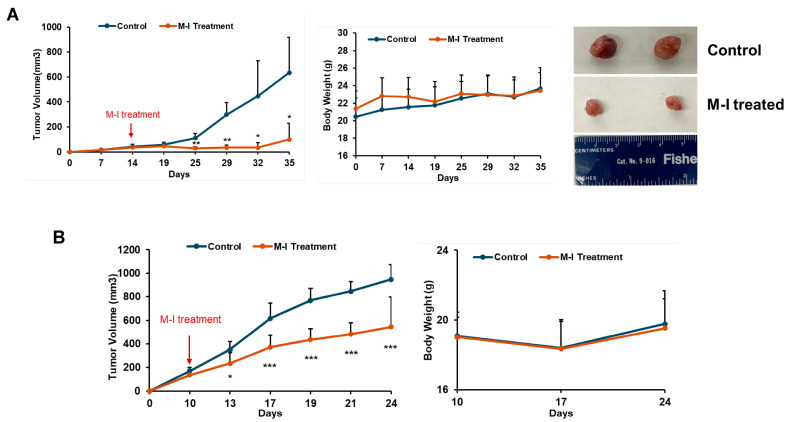
M-I treatment reduces tumor growth in TNBC mouse models. (**A**) Orthotopic xenograft model established by implanting MDA-MB-231 cells into the mammary fat pads of nude mice. Once tumor volumes were palpable, mice were randomized into two groups and treated daily with vehicle or M-I (30 mg/kg/day) by intraperitoneal (i.p.) injection. Tumor volumes and body weights were monitored throughout the study. A significant reduction in tumor volumes was observed in M-I-treated MDA-MB-231 tumor-bearing mice compared to controls. Body weights in the two groups remained similar, indicating M-I treatment was well-tolerated. Representative images showing reduced tumor size from the M-I-treated group in the MDA-MB-231 xenograft model. (**B**) Syngeneic orthotopic model with 4T1 cells established and treated with M-I similarly. A significant reduction in tumor volumes was noted following M-I treatment, without changing the body weight. The arrow indicates the starting point of M-I treatment. Data represents mean ± SD (*n* = 5 mice in each group; * *p* < 0.05, ** *p* < 0.01, *** *p* < 0.001).

Since we observed the cell growth-inhibitory effect of M-I in TNBC cells, we examined its anti-tumor effect in orthotopic breast cancer mouse models. MDA-MB-231 cells were implanted into the left mammary fat pad of nude mice. After the formation of a palpable tumor, mice were divided into two groups: untreated controls (*n* = 5) and the M-I-treated group (*n* = 5). The control group received vehicle, and the experimental group received 30 mg/kg/mouse of M-I intraperitoneally once a day until the end of the experiment. As shown in (Figure 4A) M-I treatment significantly reduced the tumor volume. We monitored mice daily for changes in body weight, food intake, and behavior. We found no significant difference in body weight between treated and untreated groups of mice (Figure 4A), as we observed previously in other cancer models [12,15]. To further examine the effect of M-I in an immunocompetent model, mouse 4T1 cells were orthotopically implanted in female BALB/c mice. Tumor-bearing mice were divided into two groups (*n* = 5/group) and treated as described above. We observed a significant reduction in tumor volume in the M-I-treated group; however, the body weight of the mice remained similar to that of the control group (Figure 4B). Mice were sacrificed, and tumors were collected for biochemical analysis. Our results suggest that in both the TNBC orthotopic xenograft and syngeneic mouse models, the M-I-treated group displayed a significant reduction in tumor volume without showing any detectable toxicity.

### 3.4. M-I Plays an Immunomodulatory Role in TNBC Tumors

**Figure 5:**
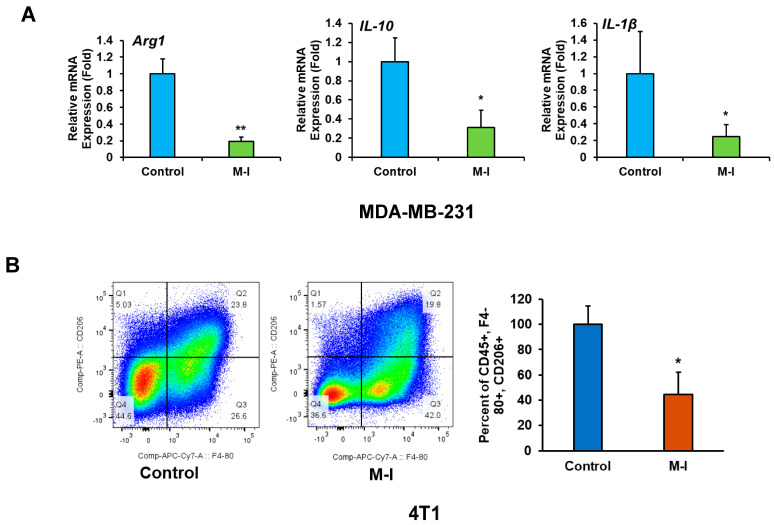
M-I treatment alters tumor-associated macrophages in 4T1 mouse tumors. (**A**) MDA-MB-231 xenograft tumor RNA from vehicle and M-I-treated mice were analyzed by qRT-PCR using specific primers targeting macrophage polarization markers (*Arg1*, *IL-10*, *IL-1β*). 18S rRNA was used as an internal control. Data indicates a significant decrease in M2 macrophage markers upon M-I treatment. The small bar indicates standard error (* *p* < 0.05, ** *p* < 0.01). (**B**) Flow cytometric analyses of 4T1 tumor-infiltrated immune cells were performed on control and M-I-treated tumors with CD45, F4/80, and CD206 immunolabelling, indicating a decrease in the CD206 cell population. The bar graph on the right shows the percentage of CD206 expression from total CD45^+^ + F4/80^+^ + CD206^+^ populations. (* *p* < 0.05).

Cancer progression is dictated by the functional state of tumor-associated macrophages (TAMs), which are the primary immune cell group in the TNBC TME. Specifically, M1- and M2-type macrophages exert contrasting effects on tumors, with either suppressive or stimulatory actions. Therefore, we investigated whether M-I plays a modulatory role in TAMs’ functional phenotypes. As M-I treatment reduces tumor volume in both orthotopic and syngeneic tumor mouse models, we became interested in investigating the alteration of TAMs following M-I treatment. TAMs are a heterogeneous population of myeloid cells that dictate the inflammatory tone of the tumor microenvironment (TME). We examined the expression level of several macrophage markers and observed a significant reduction in immunosuppressive M2 marker (*Arg1*, *IL-10* and *IL-1b*) mRNA expression in M-I treated MDA-MB-231 xenograft tumors (Figure 5A). However, we did not observe any significant alteration in M1 markers. We also examined the status of TAMs from immunocompetent 4T1 tumors. Tumor-infiltrating macrophages were stained using CD45, mouse macrophage marker F4/80, and M2 marker CD206. The ratio of CD206:F4/80 indicated that M-I-treated tumors exhibited fewer M2 macrophages compared to the control tumor (Figure 5B). The bar graph on the right shows the percentages of CD206 expression from total CD45^+^ + F4/80^+^ + CD206^+^ populations.

### 3.5. M-I Treatment Suppresses M2 Macrophage Polarization *In* Vitro

**Figure 6:**
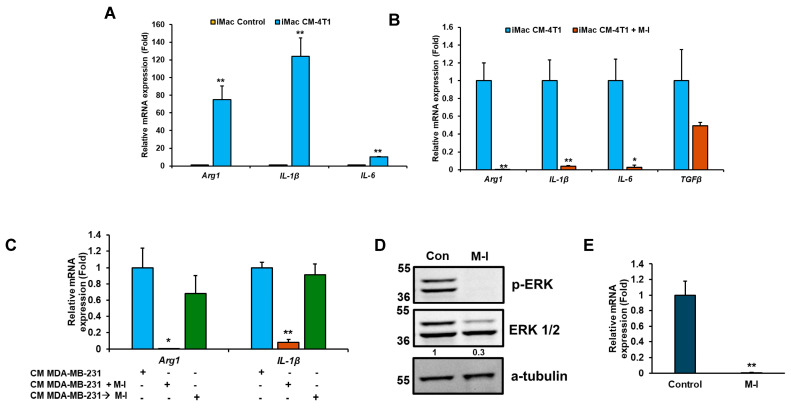
M-I treatment in TNBC cells suppresses M2 polarization of macrophages in vitro. (**A**) iMacs (immortalized bone marrow-derived macrophages) were exposed to conditioned media (CM) from 4T1 cells, and macrophage polarization markers (*Arg1*, *IL-1β*, and *IL-6*) were analyzed by qRT-PCR. 18S rRNA was used as an internal control. Data indicates a significant increase in M2 macrophage markers upon CM treatment. The small bar indicates standard error (** *p* < 0.01). (**B**) A significant reduction in the M2 marker genes (*Arg1*, *IL-1β*, and *IL-6*) was observed when iMacs were incubated with M-I-treated CM of 4T1 cells. 18S rRNA was used as an internal control. (**C**) A significant reduction in M2 markers was observed when iMacs were incubated with M-I-treated CM of MDA-MB-231 cells but not when M-I was applied directly to CM-exposed iMacs. 18S rRNA was used as an internal control. (**D**) Western blot analysis showing decreased pERK1/2 in iMacs incubated with CM from vehicle (Con) or M-I-treated 4T1 cells. A-tubulin was used as an internal control. (**E**) Reduced IL-4 mRNA expression in 4T1 cells treated with M-I was observed. 18S rRNA was used as an internal control. Data represent mean ± SD (*n* = 3, * *p* < 0.05, ** *p* < 0.01). The value shows the quantitative protein representation of Western blot band intensities normalized to the loading control using ImageJ software, presented control as 1 (arbitrarily). The uncropped blots are shown in Supplementary Materials.

To investigate the effect of TNBC cells on macrophage polarization by M-I, we conducted in vitro studies using 4T1 cells and immortalized mouse bone marrow-derived macrophages (iMacs). For this, we exposed iMacs to conditioned media (CM) from 4T1 cells. A significant upregulation of M2 markers (*Arg1*, *IL-1b*, and *IL-6*) was observed in iMacs treated with CM of 4T1 (Figure 6A), suggesting the soluble mediators from 4T1 cells alter the polarization of iMacs. Similar results observed when CM of MDA-MB-231 cells were exposed to iMacs. Interestingly, when M-I-treated 4T1 CM was incubated with iMacs, we observed reductions in *Arg1*, *IL-1b*, and *IL-6* (Figure 6B), corroborating our in vivo data. Next, we examined whether M-I inhibits M2 polarization by acting directly on iMacs or through a mechanism in TNBC cells. For this, iMacs were exposed to TNBC CM, and M-I was subsequently added to the CM-exposed iMacs. After 24 h of treatment, RNA from iMacs was analyzed for M2 macrophage marker gene expression. As expected, a significant reduction in M2 marker levels was observed when macrophages were incubated with M-I-treated MDA-MB-231 CM but not directly on iMacs (Figure 6C). These findings indicate that M-I does not act directly on macrophages but modifies mediators from tumor cells that reprogram the macrophage phenotype. To investigate the potential impact of M-I on macrophage polarization, we tested the known signaling pathways. We observed a significant reduction in ERK phosphorylation in iMacs incubated with M-I-treated 4T1 CM (Figure 6D). However, we did not detect any changes in the expression of STAT6 or STAT3 (Ray, RB, unpublished observation). IL-4 is recognized for its role in inducing M2 macrophage polarization [16]. To examine the status of IL-4, 4T1 cells were treated with vehicle or M-I, and a significant decrease in IL-4 expression was observed (Figure 6E). Together, our data strongly indicates that TNBC cells drive macrophage polarization toward the M2 phenotype through IL-4-mediated activation of the MAPK kinase pathway, which is inhibited by M-I.

## 4. Discussion

In this study, we showed that M-I treatment significantly reduced tumor growth in two TNBC mouse models without detectable toxicity. TNBC is a highly aggressive type of breast cancer, and treatment often has limited effectiveness. Many new medications that target cancer stroma demonstrate minimal efficacy in clinical trials or experience negative side effects [17]. The resistance to standard cancer treatments and a higher recurrence rate makes disease management challenging [18]. Therefore, targeted therapy alone or in combination with conventional cancer therapies could be a viable alternative approach, and there is an urgent need for safer alternative therapies. Natural compounds represent a significant and safer option and have attracted considerable interest in cancer therapy [5,6,7]. Natural compounds induce cell cycle arrest, and their role in inhibiting cancer growth has been documented in various studies [19,20]. Bitter melon extract induces cell cycle arrest and prevents the growth of prostate and colon cancer [9,21]. We previously reported that bitter melon extract or its active component, M-I, inhibits the progression of head and neck cancer by directly influencing cancer cell signaling pathways [9,12]. However, the effect of M-I on the TNBC microenvironment remains unclear. In this study, we observed that M-I inhibits TNBC cell proliferation and the expression of pSTAT3, c-Myc, and cyclin D1. These findings are consistent with previous reports that demonstrated the role of natural products in cell cycle arrest and inhibition of cancer growth [19].

The significance of the TME in the progression of TNBC has been increasingly recognized in recent years [22]. TNBC is characterized by excessive accumulation of tumor-infiltrating immune cells, including TAMs [23]. Macrophages have two functional phenotypes: M1 (activated macrophages) and M2 (alternatively activated macrophages). TAMs, most of which are anti-inflammatory M2 subtypes, are the key components of the TME, consisting of a heterogeneous population, and promote tumor growth and invasion. TAMs are an altered version of tumor-infiltrating M2 macrophages that play a crucial role in cancer progression by creating an immunosuppressive microenvironment, which has recently attracted significant attention in cancer research [24]. Targeted elimination of TAMs or the inhibition of TAM plasticity has been shown to be beneficial in various cancer types [24,25]. In glioma, pancreatic, and head and neck cancer, scRNA-seq analysis has identified both inflammatory and immunosuppressive genes [26,27,28]. In breast cancer, M2 macrophages are more abundant than in normal breast tissue [29]. Natural compounds regulate TAMs via different mechanisms [30]. Many natural compounds increase the number of M1 phenotypes by promoting polarization of M1 macrophages or reducing the number of M2 macrophages by inhibiting M2 polarization and preventing monocyte recruitment, whereas some molecules convert macrophages from the M2 to M1 phenotype. We found that M-I treatment significantly reduced the number of M2-like macrophages in TAMs, as evidenced by the downregulation of *Arg1*, *IL-10*, *CD206*, *IL-1β*, and *IL-6*. We did not observe any significant differences in mRNA expression of M1 markers iNOS and TNF-α; therefore, we focused on M2 macrophages. The total F4/80 macrophage population was also lower in M-I-treated TAMs. M2 macrophages secrete pro-inflammatory cytokine IL-1β, which is correlated with the development of esophageal squamous cell carcinoma and is elevated in metabolic disorders [31]. Matrine, a naturally occurring alkaloid found in Sophora flavescens, reduces *IL-10*, *Arg-1*, and *CD206* in TAMs in lung cancer models [32]. We found that IL-1β was elevated in TNBC tumors and iMacs exposed to the CM of TNBC cells. M-I treatment of TNBC cells and tumor-bearing mice significantly reduced IL-1β expression. In IL-6 knockout mice, infiltrating macrophages never developed a predominant M2 phenotype, suggesting that IL-6 promotes alternative activation of macrophages and that TAMs increase the tumor-initiating ability by secreting IL-6 [33]. We observed high levels of *IL-6* expression in iMacs treated with the CM of TNBC cells, which is significantly reduced following M-I treatment.

Several cytokines and signaling pathways within the TME influence M1/M2 polarization [13,17]. STAT3 or STAT6 plays an important role in M2 polarization [34,35]. However, we did not observe an alteration in this pathway in the M-I-treated TNBC cells exposed to iMacs. MAPK signaling also polarizes macrophages into the M2 phenotype [28,36]. In our experimental system, we observed that M-I-treated CM from TNBC cells significantly reduced pERK expression in iMacs. IL-4, a well-known inducer of M2 [16], was also significantly downregulated in TNBC cells following M-I treatment, suggesting that M-I may affect IL-4 signaling-mediated macrophage polarization and downstream MAPK signaling.

## 5. Conclusions

**Figure 7:**
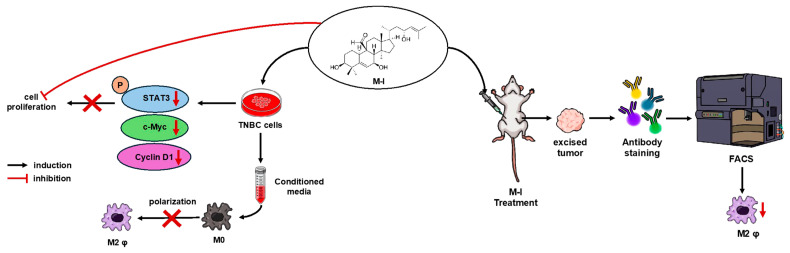
Schematic representation of M-I action in TNBC. M-I inhibits TNBC proliferation by regulating intracellular signaling. M-I reduces tumor growth without toxicity in orthotopic TNBC mouse models. M-I treatment reduces the M2 phenotype of tumor-associated macrophages.

Our study provides new insights into the M-I-mediated anti-cancer effect on TNBC through modulation of cancer cell intrinsic signaling pathways and the tumor microenvironment (Figure 7). M-I has emerged as a potential therapeutic agent for cancer because of its multifaceted effects on cellular processes [15]. Therefore, it is conceivable that M-I may exert multiple mechanisms for reducing tumor growth. M-I treatment disrupts tumor-promoting M2 TAMs in TNBC tumors, as is evident in human xenograft and syngeneic tumor models. Although our findings strongly support the therapeutic potential of M-I, further investigation into other signaling pathways that include additional immune (in immunocompetent animal models) and stromal cells that may also be involved in tumor regression following M-I treatment is needed and will be the focus of our future work. To the best of our knowledge, this is the first study describing the anti-cancer activity of M-I in preclinical TNBC models. Our current study and previous reports [12,26] report no detectable toxicity of M-I in preclinical models. Furthermore, M-I is isolated from edible bitter melon fruit. We and others have shown that bitter melon extract has no measurable toxicity in several preclinical cancer models [9]. Therefore, M-I can be tested in a Phase I clinical trial in the near future. Given M-I’s role in suppressing tumor proliferation and remodeling the tumor immune environment, M-I could be evaluated in combination with chemotherapy or immune checkpoint inhibitors to enhance efficacy in TNBC treatment. These results may also help in the development of M-I as a novel therapeutic agent to overcome the immunosuppressive effect in TNBC to improve its clinical benefits.

## Figures and Tables

**Table 1:** List of primers used in qRT-PCR.

Gene	Species	Primer Type	Sequence
*AURKA*	Hs	FP	5′-TCC TAA TAA GGT ACC TGG AGG GAT G-3′
		RP	5′-AGC TGC AAG ATC TCC GTA CGC TTA C-3′
*CDC25c*	Hs	FP	5′-GAA GAG GAC AGG TCT CTG AA-3′
		RP	5′-CTC AGT CTT GTG GTC CTG AT-3′
*CCNB1*	Hs	FP	5′-CCT GAG CCT GTT AAA GAA GA-3′
		RP	5′-TTC TGC ATC CAC ATC ATT TA-3′
*CDK1*	Hs	FP	5′-CTC CCA ATA ATG AAG TGT GG-3′
		RP	5′-GTT TGG CTG GAT CAT AGA TT-3′
*PLK1*	Hs	FP	5′-CGA TAC TAC CTA CGG CAA AT-3′
		RP	5′-CGG GAG CTA TGT AAT TAG GA-3′
*Arg1*	Mm	FP	5′-CTC CAA GCC AAA GTC CTT AGA G-3′
		RP	5′-AGG AGC TGT CAT TAG GGA CAT C-3′
*IL-10*	Mm	FP	5′-GCT CTT ACT GAC TGG CAT GAG-3′
		RP	5′-CGC AGC TCT AGG AGC ATG TG-3′
*IL-1β*	Mm	FP	5′-GCA ACT GTT CCT GAA CTC AAC T-3′
		RP	5′-ATC TTT TGG GGT CCG TCA ACT-3′
*IL-6*	Mm	FP	5′-TGT GCA ATG GCA ATT CTG AT-3′
		RP	5′-GGA AAT TGG GGT AGG AAG GA-3′
*IL-4*	Mm	FP	5′-TCT GCA TCC CGT TGT TTT GC-3′
		RP	5′-GCA CCT GTG CAT CCT GAA TG-3′

## Data Availability

All data is available in the main text. All other raw data generated in this study are available upon request from the corresponding authors.

## Supplementary Materials

The following supporting information can be downloaded at: https://www.mdpi.com/article/10.3390/cancers17142342/s1, The uncropped blots are shown in Supplementary Materials.

## Abbreviations

Arg1: Arginase 1; AURKA: Aurora kinase A; CCNB1: Cyclin B1; CDC25C: Cell division cycle 25C; CDK1: Cyclin-dependent kinase 1; CM: Conditioned media; DMSO: Dimethyl sulfoxide; ER: Estrogen receptor; ERK: Extracellular signal-regulated kinase; FACS: Fluorescence-activated cell sorting; FBS: Fetal bovine serum; G1: Gap 1 phase of cell cycle; G2: Gap 2 phase of cell cycle; HER2: Human epidermal growth factor receptor 2; i.p.: Intraperitoneal; IL-1β: Interleukin-1 beta; IL-4: Interleukin-4; IL-6: Interleukin-6; iMacs: Immortalized bone marrow-derived macrophage; M-I: Momordicine-I; MAPK: Mitogen-activated protein kinase; PI: Propidium iodide; PLK1: Polo-like kinase 1; PR: Progesterone receptor; pERK: Phosphorylated extracellular signal-regulated kinase; qRT-PCR: Quantitative real-time polymerase chain reaction; S phase: Synthesis phase of cell cycle; SD: Standard deviation; SEM: Standard error of the mean; STAT3: Signal transducer and activator of transcription 3; TGF-β: Transforming growth factor beta; TAMs: Tumor-associated macrophages; TME: Tumor microenvironment; TNBC: Triple-negative breast cancer.
