# Supplementary material for: Targeting Triple-Negative Breast Cancer with Momordicine-I for Therapeutic Gain in Preclinical Models
Source: Cancers (Basel). 2025 Jul 15;17(14):2342. doi: 10.3390/cancers17142342 (PMC12294037; doi:10.3390/cancers17142342)
Supplement: Supplementary file 1 [file cancers-17-02342-s001.zip › cancers-3744146-supplementary.pdf]

Fig. 3A

phospho-STAT3

Rabbit mAb
MW: 79, 86 kDa

CST 9145
(Tyr705) (D3A7)
1:1000

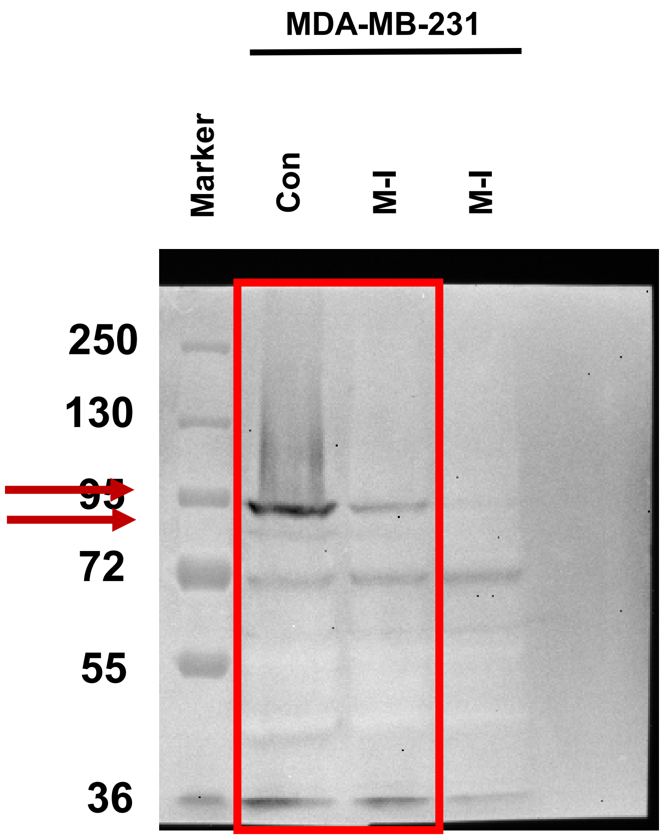

10/3/24

Fig. 3A

STAT3

Rabbit mAb
MW: 79, 86 kDa

CST-30835
(D1B2)
1:1000

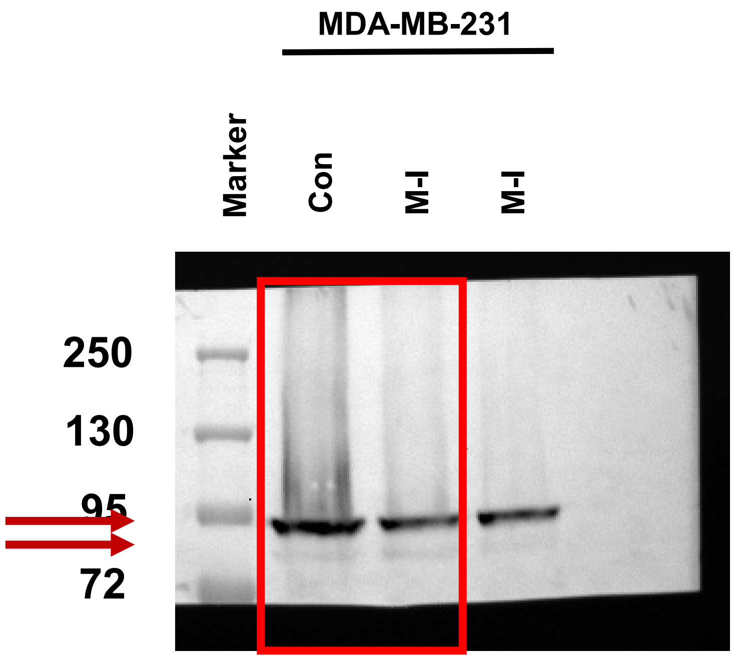

10/4/24

Fig. 3A

a-tubulin HRP

MW: 55 kDa

sc-23948
1:1000

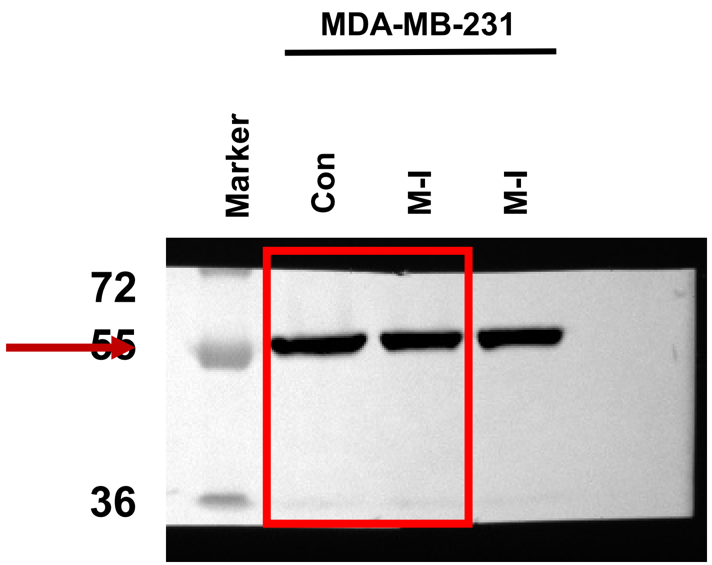

10/4/24

Fig. 3A

Cyclin D1

mouse mAb
MW: 37 kDa

sc-20044
1:500

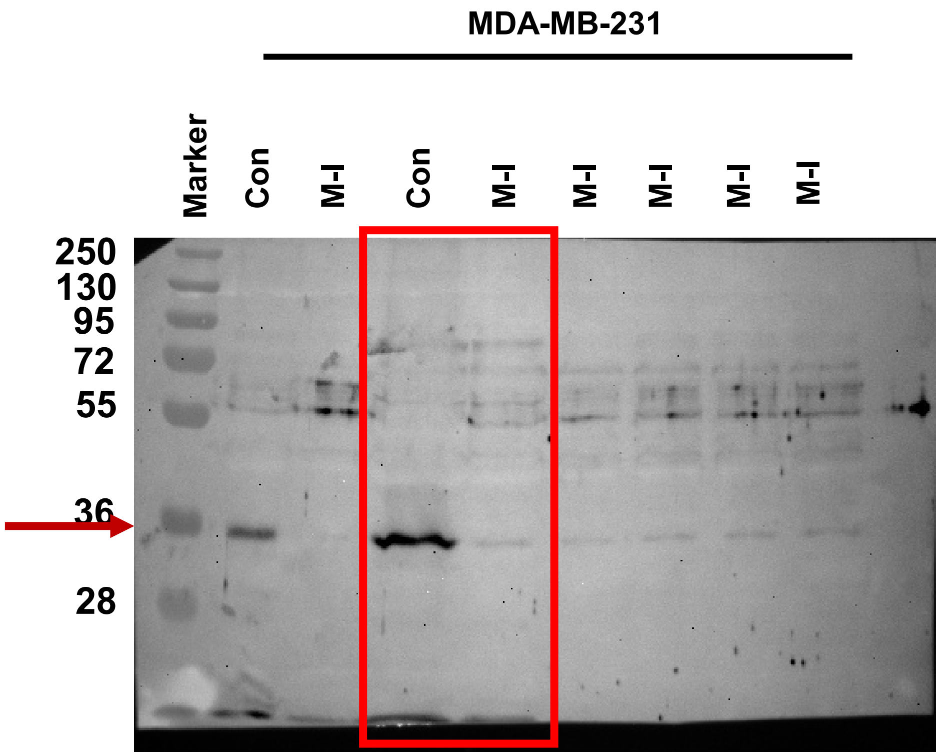

4/9/25

Fig. 3A

c-Myc

Rabbit mAb
MW: 57-65 kDa

CST-18583
1:1000

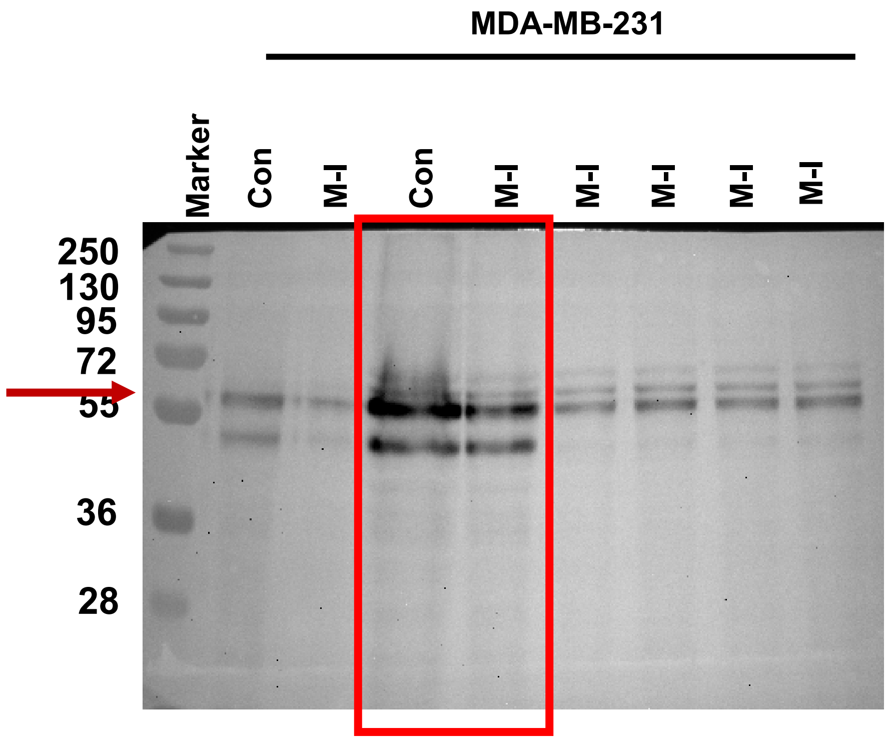

4/10/25

Fig. 3A

a-tubulin HRP

MW: 55 kDa

sc-23948
1:1000

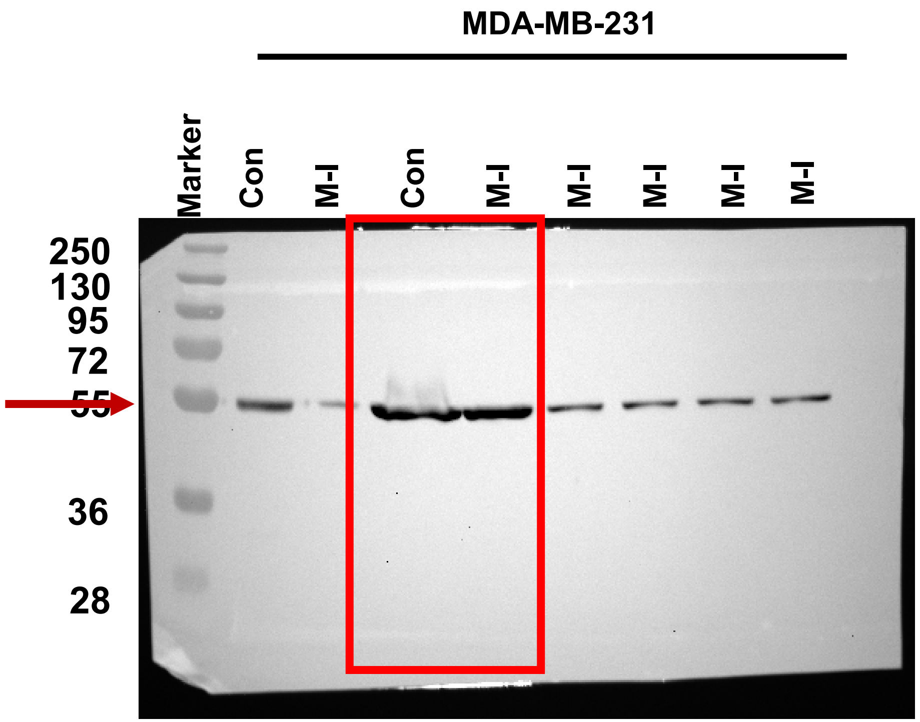

4/10/25

Fig. 3B

c-Myc

Rabbit mAb
MW: 57-65kDa

CST-18583
1:1000

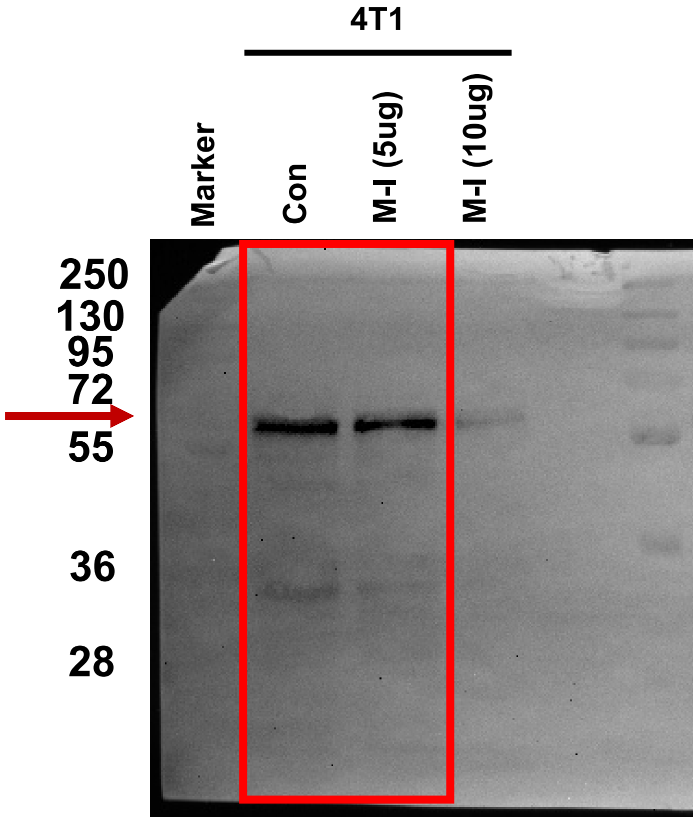

10/11/24

Fig. 3B

B-actin

MW: 43 kDa

sc-517482
1:2500

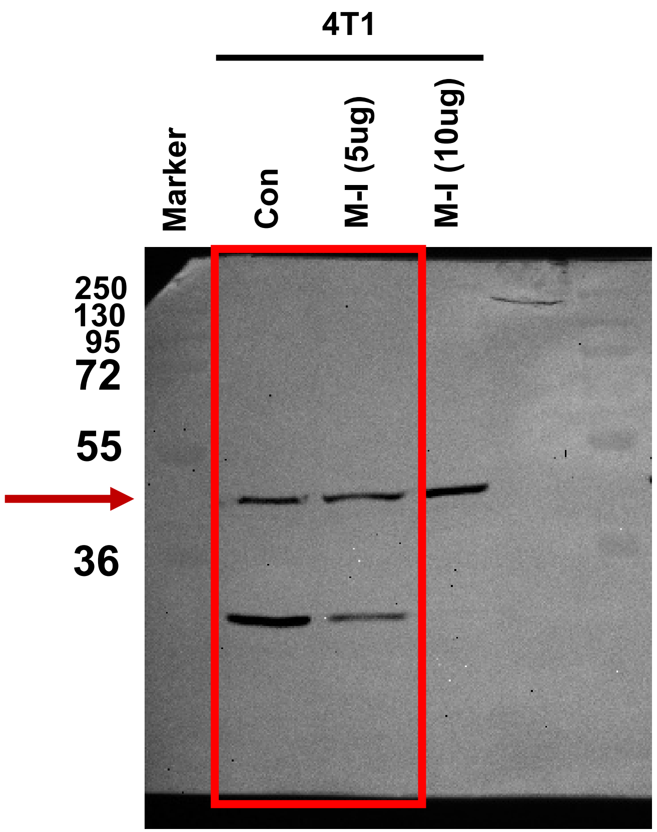

10/11/24

Fig. 3B

Cyclin D1

Mouse mAb
MW: 37 kDa

sc-20044
1:500

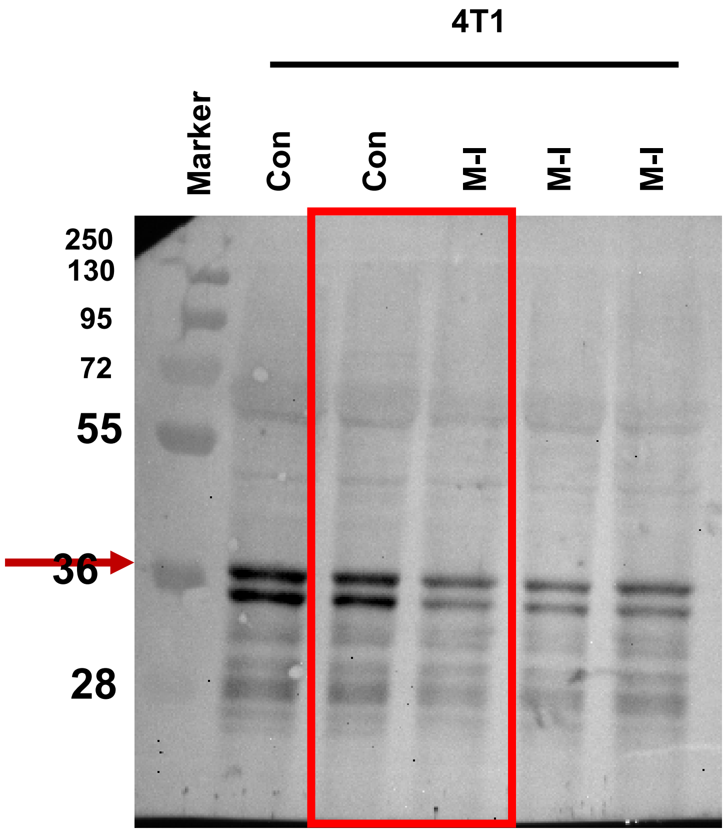

1/24/25

Fig. 3B

a-tubulin HRP

MW: 55 kDa

sc-23948
1:1000

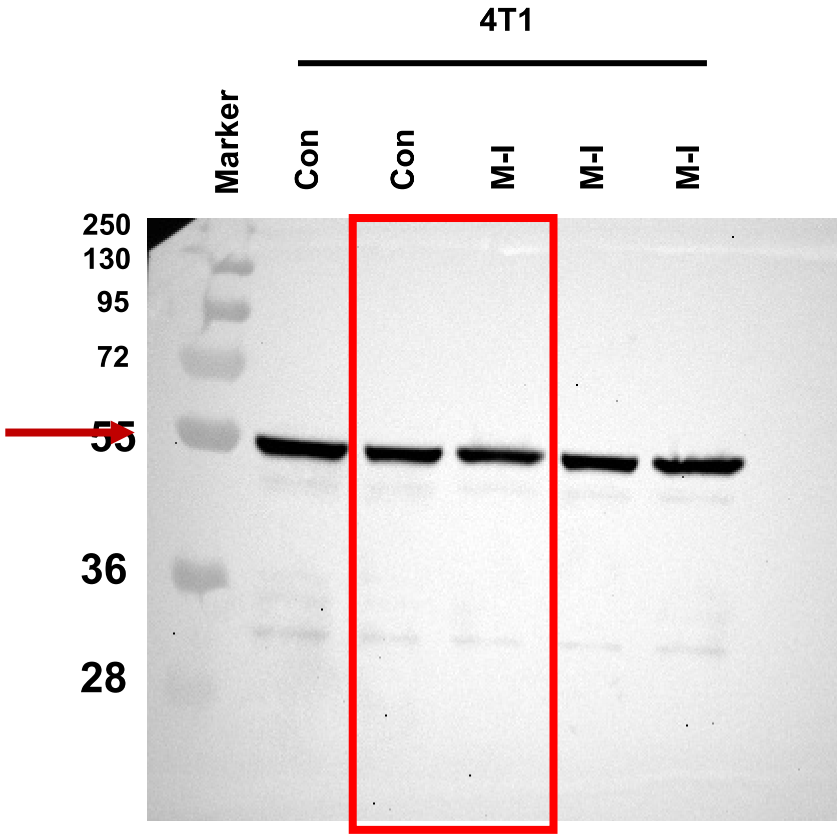

1/24/25

Fig. 6D

phospho-ERK

Rabbit mAb
MW: 42, 44 kDa

CST 4370
1:1000

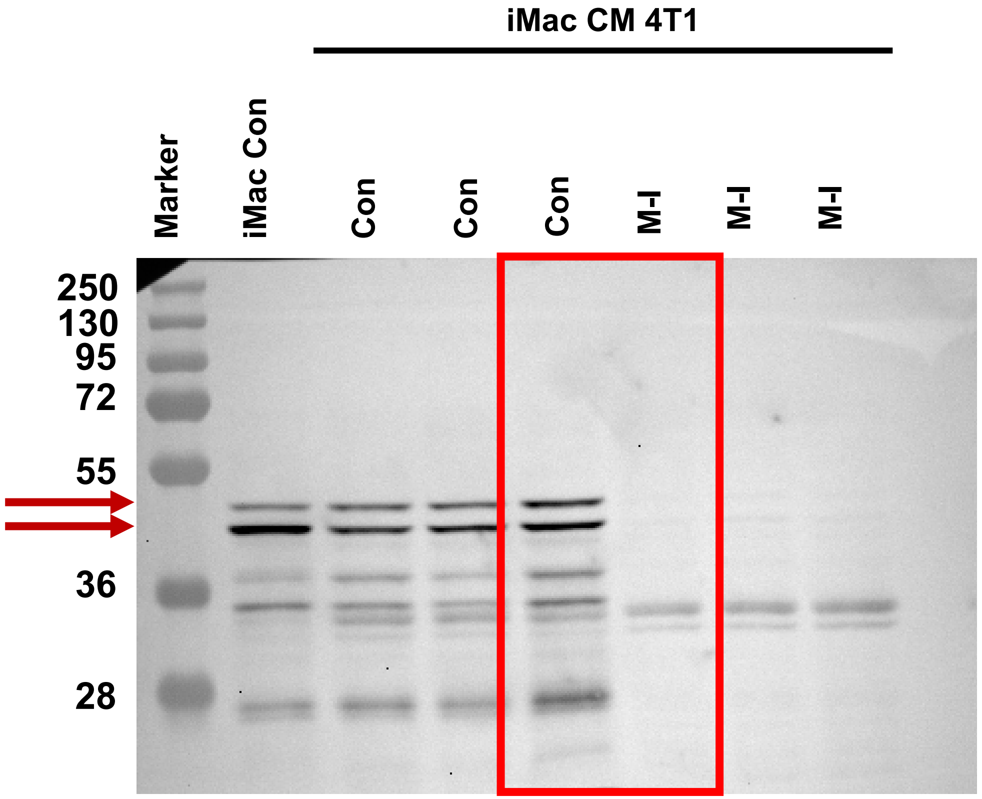

3/13/25

Fig. 6D

ERK 1/2

Mouse mAb
MW: 42, 44 kDa

CST 4696S
1:1000

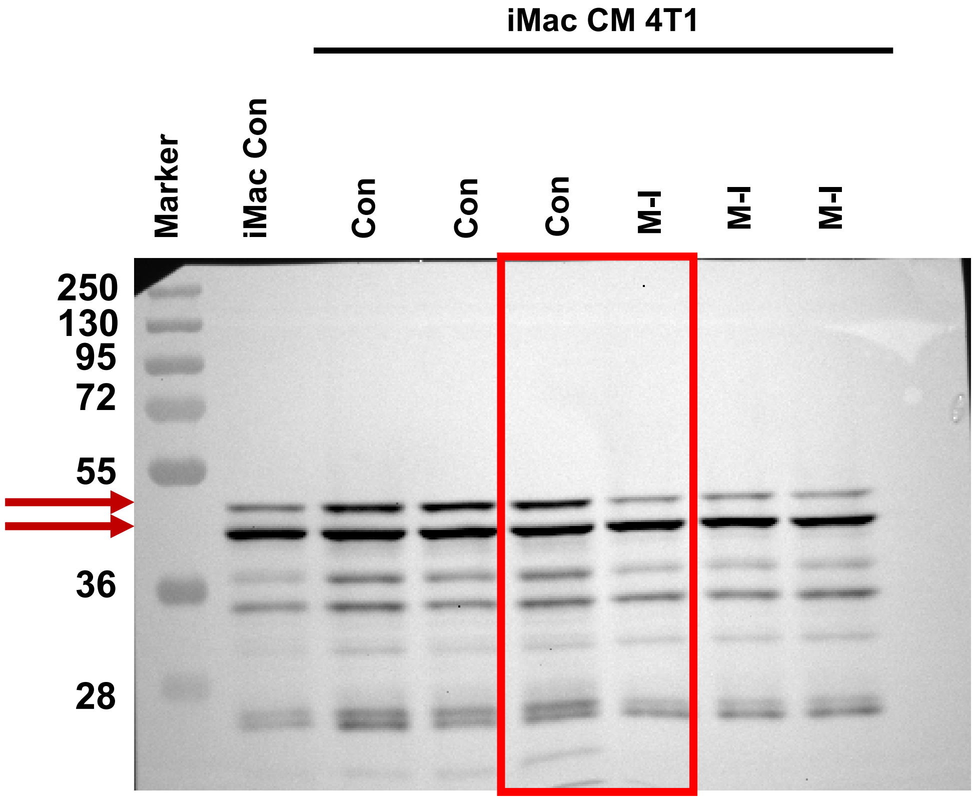

3/14/25

Fig. 6D

a-tubulin HRP

MW: 55 kDa

sc-23948
1:1000

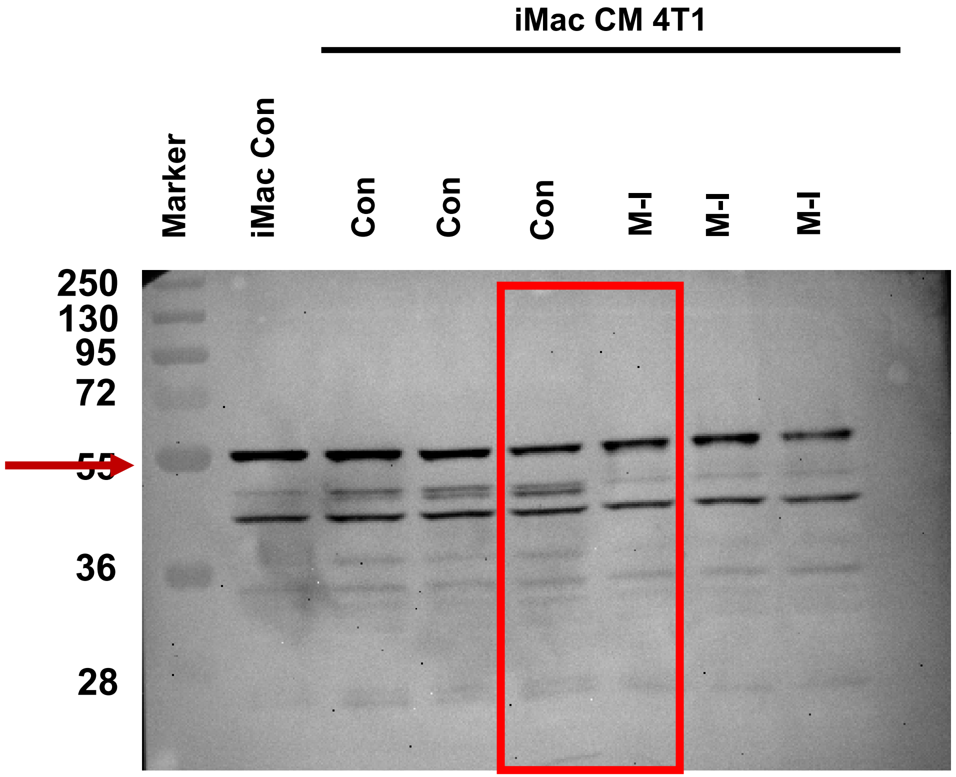

3/14/25
